# 3D Printing of Bioceramic Scaffolds—Barriers to the Clinical Translation: From Promise to Reality, and Future Perspectives

**DOI:** 10.3390/ma12172660

**Published:** 2019-08-21

**Authors:** Kang Lin, Rakib Sheikh, Sara Romanazzo, Iman Roohani

**Affiliations:** Biomaterials Design and Tissue Engineering Lab, School of Chemistry, University of New South Wales, Sydney, NSW 2052, Australia

**Keywords:** bioceramics, bioactive ceramics, 3D printing, additive manufacturing, bone tissue engineering, bioprinting, in-situ bioprinting, clinical translation, bone scaffolds

## Abstract

In this review, we summarize the challenges of the three-dimensional (3D) printing of porous bioceramics and their translational hurdles to clinical applications. The state-of-the-art of the major 3D printing techniques (powder-based and slurry-based), their limitations and key processing parameters are discussed in detail. The significant roadblocks that prevent implementation of 3D printed bioceramics in tissue engineering strategies, and medical applications are outlined, and the future directions where new research may overcome the limitations are proposed. In recent years, there has been an increasing demand for a nanoscale control in 3D fabrication of bioceramic scaffolds via emerging techniques such as digital light processing, two-photon polymerization, or large area maskless photopolymerization. However, these techniques are still in a developmental stage and not capable of fabrication of large-sized bioceramic scaffolds; thus, there is a lack of sufficient data to evaluate their contribution. This review will also not cover polymer matrix composites reinforced with particulate bioceramics, hydrogels reinforced with particulate bioceramics, polymers coated with bioceramics and non-porous bioceramics.

## 1. Introduction

While bone is known for its ability to self-heal, defects greater than a critical size hinder the natural bone-healing process and do not allow for complete healing [1,2,3]. These bone defects can arise due to trauma, infection, tumor resection, skeletal abnormalities, or when the regenerative process is compromised (such as in avascular necrosis, atrophic non-union, or osteoporosis) [4,5]. The loss of large quantities of bone tissue can easily overwhelm the body’s natural healing capacity and result in the formation of a non-union fracture [5,6]. Autografts, bones harvested from the patient’s own body, are considered the gold standard for the treatment of bone defects [7]. The key advantages of this approach include the fact that autografts are histocompatible and nonimmunogenic, and present a 3D matrix which possesses osteoconductive (stimulates bone cells to grow on its surface), osteogenic, and osteoinductive (stimulates progenitor cells to differentiate into bone cells) properties. However, autografts require a second operation at the site of harvest and therefore present high risks of inflammation, infection, and chronic pain, as well as donor site morbidity [8,9]. Besides, autograft availability can be limited in elderly or pediatric patients, patients with malignant diseases, or when dealing with a large volume of bone loss. Allografts are the second most common treatment option for bone defects, which involves transplanting donor bone tissue, often from a cadaver [10]. This method also faces limitations such as shortage of donors, high cost, need for sterilization and activation, and risk of viral disease transmission, bacterial infection, or immune rejection. Allografts are also substantially less bioactive than autografts due to the intense processes needed for sterilization [10,11]. 

During the past decades, a plethora of synthetic porous constructs, dubbed as scaffolds, has been developed to replace autografts and allografts [10,12]. Scaffolds play a crucial role in bone tissue engineering strategies to regenerate damaged tissue [13]. Scaffolds are 3D biocompatible structures that act as an extracellular matrix to support cell activity and ingrowth of newly formed tissue [14]. Interconnected pores in scaffolds allow transportation of nutrition and oxygen from the periphery of scaffolds to inner parts, migration of regenerative cells within pores, and continuous tissue formation. The vital role of scaffold pore architecture, size, and volume percentage in steering the quality and quantity of newly formed bone, vascularization, and bone remodeling are well-documented [15,16,17,18]. Among scaffolding materials, bioceramics have been traditionally a favorable candidate due to their inherent biocompatibility and bone bioactivity. Some are derived from biological sources such as demineralized bone matrix [19] and coral [20], and others are synthetic such as calcium sulfate [21], calcium silicates, bioactive glasses [22], calcium phosphate family (hydroxyapatite, β-tricalcium phosphate and α-tricalcium phosphate) [23]. Traditionally, bioceramic scaffolds are made by techniques such as gas foaming, salt leaching, freeze-drying, and the polymer template method [24]. These methods suffer from several limitations such as inflexibility and a lack of reproducibility, and more importantly, internal structure features (e.g., pore size, shape, and interconnectivity between pores) and overall shape of the scaffold cannot be precisely controlled. In the context of biological function, these structural randomnesses in scaffolds might lead to the heterogeneous distribution of cells and nonuniform tissue ingrowth [25,26,27]. 

The application of additive manufacturing technology in tissue engineering has emerged with a promise of manufacturing patient-specific scaffolds to repair the damaged tissue so that they meet the patient‘s requirements or to construct tissues and organs, on-demand. The ultimate goal is to address the need for tissue transplantations as a major clinical challenge due to the shortage of the donor. Current research on utilizing the additive manufacturing techniques in medical applications can be generally divided into five streams with the aim of developing (i) disease models, (ii) inert implants, (iii) tissue-engineered scaffolds, (iv) functional tissues and organs in-vitro, and (v) organ models for preoperative planning and educational purposes [28]. In recent years, the application of additive manufacturing in bone tissue engineering has been growing exponentially [29]. The technology has enabled layer by layer construction of bioceramic scaffolds with highly sophisticated and precise structures. This level of control in the fabrication of ceramic scaffolds is generally not feasible by using traditional methods. Physical attributes of scaffolds such as pore size, pore shape, interconnectivity between pores and porosity, and overall shape of the scaffold can be designed as a 3D model and fabricated by the printing machine [30,31]. 

Among various types of additive manufacturing techniques, stereolithography (SL), selective laser sintering (SLS), 3D printing (3DP), and direct-ink writing (DIW) are the most commonly used ones for printing bioceramic scaffolds (Table 1). These techniques can be generally classified as slurry-based and powdered-based. In slurry-based techniques such as SL and DIW, fine ceramic particles are dispersed in a liquid phase to form ink or paste, while in powdered-based techniques such as 3DP and SLS, ceramic particles are spread over a printing bed and locally bonded by laser or a binder. 

## 2. Powder-Based Techniques

### 2.1. Three-Dimensional Printing (3DP)

**Figure 1:**
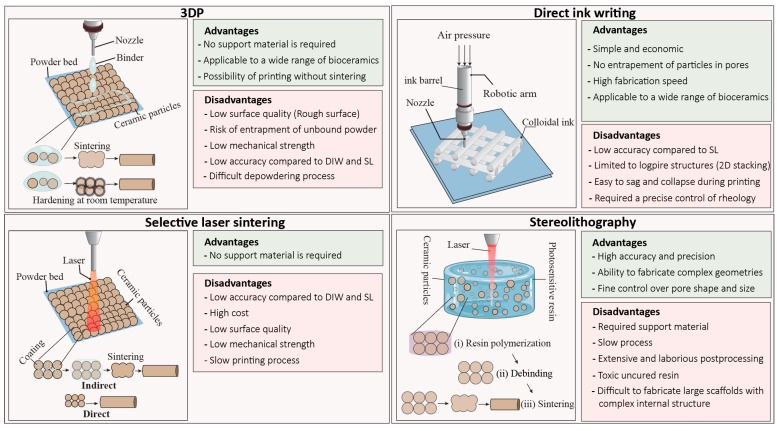
Schematic of common additive manufacturing techniques including 3D printing (3DP), direct-ink writing (DIW), stereolithography (SL), and selective laser sintering (SLS) for printing bioceramic scaffolds and their advantages and disadvantages.

Sachs et al. developed the 3DP method at the Massachusetts Institute of Technology (MIT), in 1989 for printing metallic, ceramic, and plastic parts [32]. In this method, a roller distributes the ceramic powder with a predetermined thickness onto a build platform, and a printhead sprays the binder solution (water-based or organic) on the selected region of the powder bed [31]. The binder joins the particles together either temporarily to form a green body before sintering, or permanently by promoting a reaction between particles and the binder solution (Figure 1) [33,34,35]. The movement of the printhead is according to the machine instruction language generated from the computer-aided design (CAD) file. After completion of the first layer, the build platform moves downward, and a new layer of powder spread over the previous layer then the printhead injects the binder in a specified traveling path. This process will continue until the whole part is built. After completion of the printing process, loose powders within and surrounding the green construct is removed to reveal the printed part [33]. 

In 3DP, several parameters influence the quality of printed scaffold including concentration, viscosity and volume of the binder drop, the density of powder bed after spreading of powder, powder size, morphology and surface roughness, and wettability between powder and the binder [36]. Using 3DP to construct a scaffold from a new bioceramic composition requires extensive optimization of the parameters mentioned above. The binder, either water or organic-based, binds the particles physically or initiates a chemical setting reaction [37]. Organic binders can potentially damage the machine parts, and their residue may be difficult to remove during sintering since decomposition of excessive binder leads to generating a substantial volume of gas that may lead to crack formation and damage of printed part, particularly in thin areas of the scaffold. Sintering is an essential post-treatment process to remove the organic binder and ensure sufficient mechanical properties for the printed part [38]. Sintering causes a volumetric shrinkage depending on the percentage of the binder [39]. The proper powder size and size distribution are essential for enabling a smooth particle flow over the printing bed, a well-packed powder bed, and creating fine features in the printed scaffold [40]. Finer particles help to obtain more delicate microscale features, more accurate printing, and smoother surface finishes in scaffold; however, they tend to agglomerate due to dominant van der Waals forces which results in a poor flowability and integration with the binder.

In contrast, larger-sized particles spread easier over the powder bed, and allow efficient penetration of binder into the powder bed. However, using too large particles with high flowability causes low stability of spread powder and density of powder in the printing bed [41]. Particle roundness significantly improves the flowability of powder during the printing process. A proper powder flowability allows a sufficient recoating (spreading the next layer of powder over the printed layer) for construction of a thin layer and hence a better printing resolution. To improve the flowability of a fine powder, a press-rolling technique or pre-wetting the powder has utilized, nevertheless, for the latter, the liquid content must be evaporated before injection of the binder [41]. The amount of binder released from the printhead, drop volume, and the amount of binder absorbed by the powders determine the voxel size or printing resolution [42]. The wettability between powder and the binder determines the printing resolution as well [43]. Generally, high wettability results in excessive spreading of the binder (low resolution), and low wettability lead to poor integration of particles and, hence producing a weak green body. The surface chemistry and energy of particles and the type of binder and molecular weight of organic binder determine the degree of wettability [43,44]. 

Two types of binders are commonly used in the 3DP technique, (i) acid-based (citric or phosphoric acids) and (ii) organic-based binders such as polyvinyl alcohol and poly(D, L-lactic acid) [48,76]. Acid-based binders have been extensively used for calcium phosphate powders to initiate a hydraulic setting [36,37]. In this technique, sintering is not required; nevertheless, obtained objects are extremely fragile and generally undergo postprocessing steps to become mechanically stable. 

One of the advantages of 3DP in the fabrication of bioceramic scaffolds is scalability to large sizes up to several meters [77]. The other advantages of 3DP lie in simplicity, low-cost of the technique and control over the pore size, pore geometry, and interconnectivity of scaffolds without need to design support or external platform since surrounding loose powders act as support for the print [31,43,78]. A fundamental drawback of the 3DP technique is the extensive optimization that is needed to print a quality scaffold with defined porous structure. Furthermore, choosing a suitable binder is still a challenge, and removing the binder trapped inside the small pores is difficult [79]. In comparison to slurry-based techniques, namely stereolithography, final scaffolds suffer from low resolution, rough surface finish, low density, and compromised mechanical strength. The minimum achievable pore size for a highly porous scaffold printed by 3DP technique is around 300 µm [31]. Due to the low resolution, 3DP is not a favorable technique for the processing of advanced ceramic materials. Another challenge in the 3DP technique is removing the loose powders from small pores (<600 µm) since delicate pores can easily be damaged [80,81]. In some cases, remaining loose powders are sintered inside the pores, which results in pore blockages and decreasing the overall porosity of the scaffold, which might adversely influence the tissue ingrowth. The larger the scaffold is, the risk of entrapment of loose powders in the center of scaffolds increases. In the 3DP technique, printed parts should be sintered at high temperatures except in the methods that the binder initiates a setting mechanism. Although sintering increases the mechanical strength of the print, it causes a substantial volume shrinkage that may not be necessarily uniform; therefore, it might lead to dimensional inaccuracy, residual stress, and crack formation. A wide range of bioceramic scaffolds has been fabricated by the 3DP technique including calcium phosphates family (hydroxyapatite (HA), β-tricalcium phosphate (β-TCP), α-tricalcium phosphate(α-TCP), and biphasic calcium phosphates (BCP), bioactive glasses, calcium silicates, and glass-ceramics [37,52,81,82]. Calcium phosphate powders, in conjunction with an acid binder, provide an opportunity for the printing of bioceramic scaffolds at room temperature [83]. Nevertheless, the printed scaffolds are severely weak, and they undergo a sintering stage or other postprocessing steps. Binder solutions are typically dilute phosphoric acids in a concentration range of 5 to 30 wt.% [34,36]. The most commonly used calcium phosphate powder is α-TCP with a particle size between 10 to 50 µm since it is more soluble than other types of calcium phosphates [82,84,85]. Via this technique, Klammer et al. fabricated calcium phosphate scaffolds with an identical anatomical shape to the cranial defects with the use of computed tomography [86]. They strengthen the scaffolds by additional treatment with phosphoric acid and autoclaving. Scaffolds could be drilled and stabilized with a plate and screw fixation; however, pores were substantially smaller than the optimal size required for tissue formation. Saijo et al. reported a maxillofacial reconstruction by using printed scaffolds without a sintering step. They used α-TCP powder with 10 μm particle diameter and a mixture of 5% sodium chondroitin sulfate, 12% disodium succinate, and 83% distilled water as a curing solution, which result in a rapid union in 10 patients after one year [87]. 

### 2.2. Selective Laser Sintering (SLS)

Over the past decades, SLS has emerged as a promising technique in the medical sector for printing anatomically relevant models for surgical planning and guides, customized implants, and tissue-engineered scaffolds in orthopedics and dental applications [30,88,89]. Deckard and Beaman invented SLS in 1986 at the University of Texas at Austin. In this technique, a high-power laser beam is directed on the powder bed (according to CAD file) to selectively and continuously irradiate the surface of powders to fuse them and ultimately create the 3D construct (Figure 1) [90]. 

The surrounding powders remain loose and act as support for fused particles. After scanning a layer, the powder bed moves downward, and a roller spreads the next layer of powder over the previous layer. SLS of ceramic materials can be direct or indirect [91,92]. The indirect SLS is a popular approach for fabrication of bioceramic scaffolds where the powder is coated with a sacrificial organic polymer that melts after exposure to the laser, thus binding ceramic particles together [30,92]. Subsequently, green constructs are sintered at high temperatures to produce the final scaffold [91]. The direct approach is divided into two main categories: (1) powder-based and (2) slurry-based. In this approach, simply powder heats up, and sintering takes place in-situ which does not involve complete melting of the ceramic powder, so the dimensional accuracy of the scaffold can be maintained [93]. Direct SLS of ceramics is challenging because ceramics have a high melting temperature. Although laser power can potentially generate high temperatures to initiate the sintering, which is a diffusion-based densification process, local densification of ceramic powder remains to be impractical and difficult since the laser exposure time is too short and increasing the exposure time might result in sever dimensional changes [69]. Furthermore, laser scattering by ceramic particles, laser energy consumption, and longer cooling times make this technique inefficient and not cost-effective for bioceramics. Scaffolds made by SLS technique suffers from poor dimensional accuracy, and a rough surface finish [69]. One of the reasons for dimensional inaccuracy is the conduction and diffusion of laser energy that can result in unwanted fusion of the neighboring ceramic particles to the surface of the print [41,94]. For these reasons, the quality of final products made by SLS has not yet met the industry requirements, although, for most applications in biomedical engineering it is acceptable, particularly in the fabrication of scaffolds in which surface roughness and porous microstructure are biologically desirable [95]. In the direct SLS method, the balling effect [96] is another challenge where the liquid phase turns into a spheres morphology to reduce the surface energy by forming a line of agglomerates which ultimately leads to compromised surface finish and mechanical properties. It is worth noting that print quality can be improved by the optimization of several factors, including particle size and shape, amount of binder, laser energy, and scanning speed [97,98]. SLS is essentially a more complicated technique than 3DP and robocasting techniques since densification relies on the interaction between the laser beam and particles-binder at microscale domains. This interaction will ultimately determine the dimensional accuracy, surface finish, and microstructure of the printed material, which is depended on laser power, exposure time (scanning speed) and thermal and scattering properties of particles. A large family of bioactive glasses such as 45S5, 58S based scaffolds has been printed with both direct and indirect methods [69]. The direct method is applicable for bioactive glasses and glass-ceramics since they can melt in a short time and densify through the viscous flow mechanism [69]. Nevertheless, the substantial occurrence of crystallization is the major issue in using the direct SLS of bioactive glasses. In contrast, by using the indirect approach, the rate of crystallization is more controllable. Other bioceramics such as HA, BCP have been printed mostly by indirect SLS method in combination with low-melting-point polymers or glasses [73,99,100]. A general challenge in SLS techniques remains to be low accuracy of printed scaffolds (resolution between 700 µm and 1000 µm), massive shrinkage, and low mechanical properties [101,102]. Although the dimensional accuracy seems to be better for the indirect SLS process, the direct SLS process is still an attractive technique for the fabrication of bioactive glass or silicate-based bioceramic scaffolds, since no additional post-processing step is required, which reduces the fabrication time and costs [30]. Currently, only a few SLS products have been used in clinical applications mostly limited to plastic models as surgery guides or as bone scaffolds used in vitro and in vivo in animal models. 

## 3. Slurry-Based Technology

### 3.1. Stereolithography (SL)

Among current 3D printing techniques of bioceramics, SL offers the highest precision in creating complex structures with control over creating a fine internal architecture down to the micrometer scale with a high-quality surface finish [103,104,105]. SL has been extensively applied to the fabricate of bone scaffolds and dental components from bioactive glasses, HA, β-TCP, zirconia, and alumina [59,106,107]. As one of the oldest technologies, SL was developed by Chuck Hull, the “father of 3D printing” in 1986, primarily to manufacture polymeric structures [108]. In the SL technique, an ultraviolet (UV) laser selectively solidifies a photosensitive liquid resin layer-by-layer to build a 3D construct. When the irradiation of a layer finishes, the resin bed is moved downward or upward by the thickness of a layer, depending on the machine build (top-down or bottom-up), once the fresh resin covers the solid layer, it hardens by exposure to UV radiation. This process continues until the completion of the final construct (Figure 1) [103,109]. The same principle can be applied to use SL for printing ceramic materials. Graham and Holloran replaced the resin-based system with a slurry system that mainly consisted of a suspension of ceramic particles with micro/nanometre size, light-sensitive monomers, and a photoinitiator which become solidified by UV laser through the photo-polymerization mechanism [110]. Other components are dispersant agents to help stabilization of ceramic particles and prevent particles from agglomeration and settling [111]. Ceramic slurry should have long-term stability and suitable rheological behavior to enable a smooth flow for printing and homogeneity of the printed part. In terms of viscosity, the slurry has to be ideally comparable to the resin (<3000 mP·s) [103,112]. However, this is somehow impractical, since a high content of ceramic particles in resin is necessary for reducing the shrinkage during debinding and sintering [113]. Most slurries used in SL are non-aqueous and based on resin acrylamide because aqueous-based suspensions result in weak printed structures [113]. One of the immediate differences between traditional SL and ceramic SL is the contribution of scattering phenomena due to the addition of ceramic particles to light-sensitive monomer [110,113]. The ceramic particles scatter the UV light, thus reducing cure depth, resolution, and increasing the printing time. Generally, the curing rate decreases with increasing refractive index ratio between ceramic particles and the organic content, which becomes even slower for larger particle size [113,114]. Therefore, a smaller particle size is preferable for SL technique since they have a better scattering property. Nevertheless, cure depth can be controlled by adjusting the power of the laser, scan speed, and exposure time [115,116]. 

Ceramic particles are inert to UV laser, and polymerization occurs only in the organic monomer phase. Therefore, the key parameter is to ensure uniform distribution of ceramic particles in the organic network until the entire 3D structure is built up [117]. The printed structure has to be calcined to remove the organic components (also called the debinding stage) and subsequently sintered at high temperatures to fuse the ceramic particles [118]. The main advantages of SL are versatility, freedom of design to create constructs with the scale of several microns to decimetre, and high dimensional accuracy. Therefore, SL has the potential for manufacturing ceramic implants based on patient-specific needs.

On the other hand, the main disadvantage of SL is the need for supporting material that will require integration with a temporary support model to make the printing of overhangs and cantilevers possible. The detachment of these supports is challenging, particularly for complex architectures. Various parameters influence the success of SL of ceramic scaffolds including volume ratio of ceramic powder to organic components, ceramic particle size and distribution, chemistry and concentration of monomer, UV power, exposure time and wavelength, viscosity of the slurry, the interaction between ceramic particles and organic components, and refractive index difference between powder and monomer [119]. 

Debinding, removing the organic components from the print by increasing the temperature (typically up to 550 °C) to decompose and vaporize the organic phases, is the most important postprocessing step [118]. Each organic component has a different decomposition temperature, and depending on the volume of organic components, a substantial reduction in weight and dimension of the print occurs. After the debinding process, the polymer matrix that holds ceramic particles burns out, and a structure containing loosely packed ceramic powder is left. Hence, the rate of debinding should be carefully controlled to prevent the formation of crack during diffusion and evaporation of the pyrolyzed organic components [41]. Typically, debinding of a scaffold with a higher surface area to volume ratio and smaller dimension is more successful. The debinding process depends on several parameters such as ceramic particle size and distribution, the composition of organic components, solid content in the slurry, and the dimension of the print. After debinding, printed parts are sintered at high temperatures to obtain a dense microstructure. The printed parts experience a large volume shrinkage during debinding and sintering steps in which the extent of shrinkage is proportional to the amount of ceramic powder in the slurry. To minimize the shrinkage, and increase the accuracy of the shape, a higher solid should be introduced into the slurry. However, a high particle content may result in the formation of crack during the debinding process due to hindering the diffusion of gasses from reaching to the free surface of the sample which builds up a considerable pressure inside the printed layers. 

### 3.2. Direct Ink Writing (DIW) 

Direct ink writing (DIW) also knowns as micro-robotic deposition, robocasting or direct printing involves depositing a water-based colloidal suspension (ink) with high volume content of ceramic powder that has a capability of maintaining its physical integrity and support its weight without deformation upon extrusion from a small nozzle (Figure 1) [120]. In this technique, developed by Cesarano in 1997, objects are built up layer by layer via extrusion of the ink through a moving nozzle controlled by a robotic arm [68,121,122]. 

Compared to other techniques, DIW is more economical and faster where the entire process, including fabrication, drying, and sintering can be completed in less than a day [121]. The challenge, however, is to develop a ceramic ink that is suitable for the deposition process with a shear-thinning property and ability to retain its shape after extrusion. Ink should contain a substantially high content of ceramic powder compared to the liquid phase to yield a high stiffness and at the same time, show a fluid-like consistency to flow through a small nozzle [123,124]. The solid content of ink is typically between 50 and 65 vol.%. This high solid content prevents the formation of a crack in scaffold during drying and enables the ink to retain its shape after deposition [125]. Since this level of solid loading is relatively high, ink viscosity and stability become sensitive to slight changes of solid loading, and therefore, the addition of ceramic powder to liquid phase should be done in a controlled manner [124]. The ink should contain a high volume percentage of the ceramic powder and have a tailored viscoelastic property to facilitates a continuous flow through a nozzle with small diameter while maintaining a strength that allows it to span large gaps without bending [120,124]. Ceramic particles that are agglomerated, with a rough surface and irregular morphology are not favorable. Particles have to be ideally monosized (having a narrow size distribution) with a smooth surface. Presence of large particles can result in clogging the deposition nozzles, and small particles tend to agglomerate and destabilize the ink. The surface of particles should be modified with dispersing agents (surfactants) to prevent agglomeration in the liquid phase and to obtain a high solid loading [120]. The typical dispersing agents use electrostatic, steric, or electrosteric forces to promote colloid stabilization. Another component of the ink is binder or viscofier; they are essentially polymer chain molecules that adsorb to the surface of particles and link the particles together, modify viscoelastic properties of ink, and help the structural integrity during drying [126]. One of the popular binder used in DIW technique is methylcellulose-based binders that are nonionic and promote the shear-thinning property [127]. Cellulose fibers align with the direction of the shear force hence reducing the ink viscosity. The last step in the preparation of ink is the flocculation or destabilization step, in which the viscosity of the ink increases through the segregation of particles from the organic network [126]. Flocculation can be initiated by adding salt solutions, oppositely charged polymer solution to neutralize the charge on the dispersant, or decreasing the pH [125,128]. This step should be performed carefully because the ink can become irreversibly destabilized. The challenges in robocasting are printing multi-material structures, frequent incidents of nozzle clogging, laborious ink optimization, high sensitivity of ink to processing parameters, and ‘cake formation’ due to consistent extrusion pressure on the ink and separation of particles from the liquid phase. The typical defects in scaffolds made by DIW can be generally classified into the formation of gap and necking, layer dragging, incomplete corners, and globular region. DIW has been one of the most studied techniques in the fabrication of bioceramic scaffolds for bone tissue regeneration. A wide range of structures from solid monoliths to complex porous bone scaffolds have been fabricated and test in-vitro and in-vivo [17,66,121,129,130,131]. One of the attractions of the DIW technique is its versatility in printing almost any types of bioceramics ranging from calcium phosphates, bioactive glasses to calcium silicates and newly formulated bioceramics, control over lattice arrangement, pore size, and pore orientation as well as low cost of operation [68,123,124,131].

## 4. Barriers to Clinical Translation and Current Challenges 

**Figure 2:**
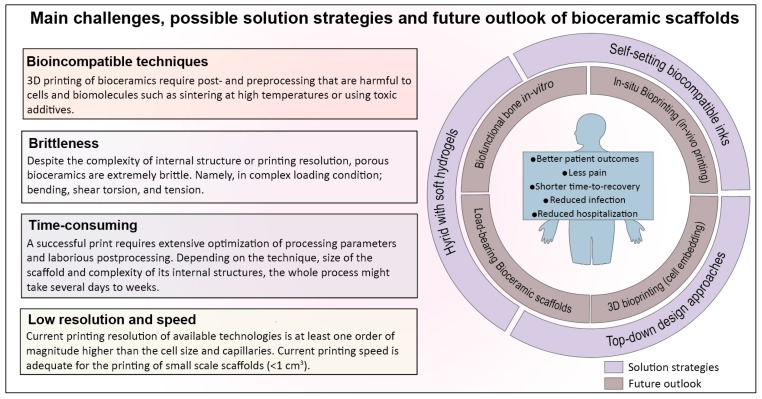
Schematic of the main challenges in additive manufacturing of bioceramic scaffolds, proposed solution strategies, and future outlook where the new research might overcome those challenges.

Historically, bioceramics have been the most favored ‘synthetic solution’ for the repair of bone defects [132]. In addition to the reconstruction of bone defects, there are other clinical needs such as spinal arthrodesis, chondral injury, and periprosthetic joint infection that can be addressed by bioceramics. In recent years, strategies to synthesize new formulations and techniques of producing bioceramics are rapidly evolving with an ultimate aim to replace or minimize the need for autografts [89,133]. A wide range of premade synthetic materials made from bioceramics in the form of blocks or granules has been developed with a global market value of US$2.7 billion in 2017 which is expected to reach US$3.9 billion by 2025. In the past two decades, additive manufacturing has emerged as a technology with a promise of patient-specific solutions and generating synthetic bone grafts with complex anatomical architectures [89]. Current research in 3D printing of bioceramics for bone regeneration is primarily focused on porous biodegradable and bioactive scaffolds with desirable architecture (pore size, geometry, and shape), chemistry and microstructure that trigger a regenerative response in interaction with host cells, aiming to promote osteogenesis and vascularization, particularly for the treatment of large bone defects. While there are few scattered encouraging outcomes from pilot studies and clinical trials on dental and maxillar and mandibular bone defects, the challenges remain to be the translation of 3D printed bioceramics into the clinic and fulfilling the promise of patient-specific solutions [134]. As discussed previously, the most popular and well-studied 3D printing techniques of bioceramics are SL, SLS, DIW, and 3DP, with advantages and drawbacks that await solutions. Some of these challenges are attributed to the nature of bioceramics, i.e., inherent brittleness, the necessity of high temperature for sintering, and the rest are related to the limitation of printing technique, preoperative planning, and postoperative complications, in which we will elaborate more in the following section (Figure 2). 

### 4.1. 3D Printing Techniques for Bioceramics Are Time-Consuming and Sensitive to Post/Preprocessing Steps

In all of the 3D printing techniques for bioceramics, numerous processing parameters have to be optimized to print a scaffold successfully. Thus, finding the best printing condition becomes even more challenging for a large-sized scaffold with a fine internal structure. Depending on the complexity of the architecture and size of the scaffold, postprocessing (e.g., debinding step, removing the loose powder or remained slurry from pores and sintering), might take several days to weeks before obtaining the final product. For example, to prepare an ink with proper viscoelastic property in DIW method, ceramic particle size and distribution, particle shape and surface topography, the concentration of solids and additives (dispersant, binder, and coagulating agent), drying procedure and method of precursor homogenization have to be carefully controlled [120]. A small variation in the concentration of additives or ratio of solid to liquid results in obtaining a ‘soft ink’, which deforms plastically upon extrusion and cause printed layers to merge, or a ‘stiff ink’ with a poor shear-thinning property that does not flow properly and requires an excessive pressure to be extruded from the nozzle. In a typical ink preparation routine, when an anionic dispersant is used to disperse particles in aqueous solution, a cationic agent is usually required to neutralize repulsive electrostatic forces and increase the stiffness of the ink. The excessive addition of cationic agent will lead to irreversible destabilizing of the ink and permanent segregation of particles from the polymer network. Furthermore, those parameters influence dimensional variations, probability of crack formation during drying, and the mechanical properties of the scaffold after sintering.

### 4.2. Bioceramic Scaffolds Are Brittle, Regardless of the Printing Technique or Complexity of Internal Structure

A fundamental challenge in clinical translation of 3D printed bioceramic scaffolds, particularly for load-bearing applications, is the brittleness. Regardless of the technique used to fabricate the scaffolds and the resolution of printing, ceramic scaffolds are inherently brittle and lack the mechanical stability at highly porous form [135]. Low strength and high brittleness make the handling of bioceramic scaffolds difficult even after sintering at high temperatures. The question is, can we utilize additive manufacturing techniques to design a tough ceramic structure that is resistant to crack propagation? In many natural materials such as nacre or bone, high toughness is achieved through a hierarchical arrangement of organic (tough phase) and inorganic (stiff phase) components at different length scales from nano to micro and macro, but such structures are extremely difficult to mimic and fabricate [136]. Nevertheless, these solutions seem to be impractical to be implemented in the 3D printing of bioceramics. Firstly, bioceramics scaffolds are required to be sintered at high temperatures; therefore, they cannot be integrated with biopolymers while printing, and secondly, the current resolution of printing techniques is limited to several microns that do not allow nanoscale control in fabrication. One of the possible solutions might be to utilize a top-down approach and design a cage made from a tough polymer as a mechanical shield in a way to accommodate the ceramic scaffolds and protect them from failure loads. 

### 4.3. Prefabricated Bioceramic Scaffolds often Fail to Match with the Anatomical Shape and Size of the Defect

During the past decades, a plethora of bioceramic scaffolds have been used in the treatment of long bone fracture, fracture nonunion, infections, hand enchondroma, and metacarpal fracture, benign tumors and cysts, or to promote bone formation in spinal fusion, open-wedge tibial osteotomy, oral/periodontal procedures, cranioplasty, vertebroplasty, and kyphoplasty. Most of the scaffolds are in a prefabricated form; however, the size and shape of the final defect to be filled may not be accurately known before the surgery. The shapes of bone defects caused by trauma, tumors, or disease are often irregular. There is also an unavoidable need for intraoperative debridement before implantation of such scaffolds, which makes the patient-specific prefabricated scaffold fail to match with the shape and size of the actual defect [137]. Furthermore, none of these scaffolds are vascularized, nor can be formed/molded to match the defect site. Besides, utilizing a 3D printer to produce prefabricated bone scaffolds in operation theatre or before surgery presents several drawbacks. These include (but not limited to) a high cost of equipment, possible deviation between computer 3D model and the defect anatomy, additional preoperative planning time, increased patient radiologic exposure for imaging, complex coordination between engineers and surgeons, additional operating time, challenging sterilization procedures, inflexible with respect to the changing condition of the wound and filling irregular defects [138]. According to a systematic review by Martelli et al. for 3D printing applications in surgery, over 20% of the studies (34 cases) reported an unsatisfactory accuracy in the prefabricated objects. The dimensional inaccuracy was due to the limitation of resolution in initial computed tomography (CT) and magnetic resonance imaging (MRI) scans, and the accuracy of the 3D printing technique [138]. 

A new strategy to address limitations associated with prefabricated scaffolds is the in-situ printing of scaffolds directly into the wound area. O’Connell et al. developed a hand-held printer (biopen) for osteochondral reconstruction, which allowed the surgeon to directly construct a desirable 3D scaffold into the defect [139]. Compared to conventional printers, a handheld bioprinter is easier to use and sterile and offers greater flexibility or precision to ‘fill in’ the irregular and hard to reach bone defects. Overall, in-situ bioprinting seems to minimize issues associated with using prefabricated scaffolds, enable surgeons to anticipate problems that might arise during the procedure, and reduce the preoperative planning time and cost. For bioceramic-based scaffolds, however, in-situ printing demands a new ink formulation, one that does not require high temperatures for densification, allows hardening in physiological condition without generating heat or toxic by-products and proper viscoelastic property. 

### 4.4. Bioceramics and Their Printing Techniques Are Incompatible in Integration with Cells and Biomolecule during Printing: 3D Bioprinting versus 3D Printing

3D bioprinting, a promising technology in regenerative medicine, is the adaptation of 3D printing techniques to create cell-laded constructs with biological functions similar to native tissue [140,141,142]. This approach utilizes a bio-ink composed of cells, biomolecules (e.g., growth factors) and a hydrogel that acts as an extracellular matrix. Bioprinting allows precise deposition of the bio-ink layer-by-layer with spatial control over the placement of the functional components. Hydrogels are the dominant choice of biomaterials in 3D bioprinting techniques, and there is no available data on bioceramics used as the main component of ‘bio-ink.’ While bioceramics are bone bioactive, they require high temperatures to form a mechanically stable 3D construct, which makes it impractical to be integrated with biomolecules or cells during the fabrication process. Conventionally, bioactive molecules and cells integrate with prefabricated scaffolds through surface adsorption and a simple post cell-seeding [29]. This limitation can be overcome by developing bioceramic inks that harden in aqueous media under the physiological condition with a proper viscoelastic property and setting time to allow layer by layer construct of 3D structures. The ink components and hardening mechanism should not be toxic to cells or detrimental to the functionality of biomolecules, and as printed scaffolds should have enough mechanical strength to provide a matrix for cell growth and proliferation. 

### 4.5. Printing Resolution, Vascularization Strategies, and Practical Challenges in Adoption of Additive Manufacturing in Clinical Practice

Bone scaffolds are expected to hold a growth-directing architecture for cell migration and proliferation and provide bioinstructive cues for cells that ultimately lead to tissue regeneration [143]. Scaffolds should have a comparable architecture and biofunction to bone tissue in different scales, with desired physicochemical properties such as surface roughness, microstructure, and porosity. Currently, the realization of biofunctional scaffolds is still at an early stage, and continuous improvement is required to overcome the limitation of technology in various clinical conditions. One of the biggest challenges in scaffold-based bone reconstruction is a lack of technology to integrate a vasculature system with bioceramic constructs. Diffusion has a limitation of 100–150 µm; therefore, the formation of a vascular network in a thick construct (>150 µm) is essential to provide sufficient nutrient, oxygen, and waste removal for the viability of newly formed tissue, particularly in the early stages of implantation so that endothelium starts homoeostatic balance. Lack of vascularization in large bone defects results in necrosis and hampers bone formation in the center of the scaffold [144]. One approach is to directly mix angiogenic factors with prefabricated scaffolds; however, this method is complex, unreliable, and difficult to control. None of the current 3D printing technologies for bioceramics can incorporate angiogenic factors into scaffolds during fabrication, nor they can print vasculature network within the scaffold pores. One reason is the necessity of invasive postprocessing procedures to produce mechanically stable ceramic scaffolds, and the second reason is that the current 3D printing resolution is lower than vessels diameter. Until now, printing resolution is generally one order of magnitude higher than the cell size and capillaries; besides, the speed and reproducibility of the printing process should be significantly improved. 

3D printing technology emerged with a promise to shorten the operation time for hard tissue regeneration and increase the efficiency of surgical procedure, meaning a shorten the exposure time of wound, less anesthesia dosage, and in turn, minimize the chance of infection and surgical complications [138]. But to accomplish the required accuracy and depend on the size of the scaffold, the preoperative designing and planning time can be substantially prolonged.

Moreover, considerable involvement of the surgeon in the predesigning process greatly increases the cost of the technology. Post-cell-seeded is even more complicated for 3D printed bioceramic scaffolds, as it requires the development of a scaffold that matches the mechanical requirement and to overcome technology limitations at the same time. The resolution, speed, and material compatibility are the main challenges of the current additive manufacturing technology, which require solutions before the further advance in clinical applications. Currently, within the available techniques, SL offers the highest accuracy and resolution of the final scaffold with complex structures, but it requires extensive and time-consuming optimization and high investment for the equipment, which has limited its widespread use. Besides technical issues, there are other obstacles ahead of translation of printed scaffolds such as stringent regulatory requirements and lack of evidence for biological performance in clinical surgery.

## 5. Future Perspective 

Three-dimensional printing techniques have emerged to create complex constructs for tissue regeneration and repair with a great promise of patient-specific treatment. Hydrogels among the different types of biomaterials have been the most popular choice since they can be co-printed with cells and bioactive molecules to generate a biofunctional 3D construct. Bioprinting has facilitated the transition from using post-cell-seeded scaffolds to the incorporation of the bioactive components during the printing to produce constructs that have controlled biofunction and biomimetic structure. Recently, in-situ printing has been developed by directly printing hydrogel-based constructs in-vivo, where the natural body environment provides necessary cues for tissue regeneration. For bioceramic scaffolds, this transition has been slow due to various challenges that were discussed in the previous section. We speculate several pathways in which new research might overcome some of these challenges (Figure 2); (i) development of bioceramic-based inks that set in aqueous media which allow printing of bioceramics at room temperature and physiological condition without using toxic components or sintering. Bone is an organic-inorganic nanocomposite material where its main cells are embedded in a highly mineralized matrix in close interaction with calcium phosphate nanoparticles. Therefore, it is plausible to generate bioceramic inks that can accommodate embedding metabolically active bone cells which will potentially enable in-situ bioprinting of a bioceramic-based scaffold in a damaged area. (ii) Integration of 3D printed bioceramic scaffolds with soft hydrogels [145,146]. This strategy would enable utilization of multiple materials to build biomimetic bone tissue constructs at a clinically relevant scale with comparable bio-functionality to the bone; moreover, structures with vascularized networks can be generated in vitro. (iii) Implementation of top-down approaches to design modular cages to accommodate prefabricated porous bioceramics, regulate stress and strain within the scaffold, and protect them against complex loading in vivo, particularly at load-bearing sites. The advantage of additive manufacturing techniques compared to the conventional approach is the ability to design and construct scaffolds with patient-specific geometries and controlled macro-micro-structure to facilitate tissue regeneration. Among different types of biomaterials, 3D printing of bioceramics is the most challenging because of their inherent brittleness and high melting temperature, and their current techniques require extensive and time-consuming optimization. Additive manufacturing techniques for bioceramics are still in infancy, where the craniomaxillofacial reconstruction surgery is the only area where most of the clinical translation has occurred. Still, constructing precise scaffold with features less than 100 μm remains challenging, fabrication of large-sized scaffolds with intricate internal structure is almost impossible due to cracking during postprocessing stage, or the entire process from pre-pre-processing to obtaining the final scaffold might take days to weeks. Despite all the advances in additive manufacturing techniques for bioceramics, there are still major gaps in this field relative to dimensional accuracy, time-consuming optimizations, invasive postprocessing steps such as sintering that prevent integration of scaffold with living cells and growth factors or biopolymers during printing, and nanoscale control during fabrication. 

## Figures and Tables

**Table 1:** Various bioceramic compositions printed by common additive manufacturing techniques.

Printing Technique	Composition	Densification Method	Strut Size (µm)	Pore Size (µm)	Porosity (%)	Finish Quality ^+^	Reference	Pore Shape
**3DP**	CaSO_4_/HA/β-TCP	Setting reaction	1000 –2000	1000–2000	50	E	Zhou et al. [45]	Cubic
**3DP**	HA/β-TCP	1200 °C	NA	100–600	65.3	E	Strobel et al. [46]	Spherical
**3DP**	α-TCP	Setting reaction	1500	844	59.2	E	Castilho et al. [47]	Spherical to Cubic
**3DP**	α-TCP/CaCO_3_	Setting reaction	1250	300	68	E	Castilho et al. [48]	Cubic
**3DP**	β-TCP, β-TCP/ZnO/SiO_2_	1250°C	1000–2000	400–700	30–50	E	Feidling et al. [49]	Cubic
**3DP**	HA	1250 °C	330	450	-	F	Seitz et al. [50]	cubic
**3DP**	HA and *β*-TCP	1250 °C	900	500	40	F	Warnke et al. [35]	Cubic
**3DP**	Ca_4_(PO_4_)_2_O	Setting reaction	200	750	40	E	Mandal et al. [51]	Cubic
**3DP**	α/β-TCP/Ca_4_(PO_4_)_2_O	Setting reaction and 1100 °C	1000	500	56–61	E	Vorndran et al. [52]	Cubic
**3DP**	CaSiO_3_ precursors	900 °C	~1000	~2000	48–53	F	Zocca et al. [53]	Cubic
**SL**	45S5 Bioglass^®^	1000 °C	540–1000	1000	50	B	Tesavibul et al. [54]	Cylindrical cellular
**SL**	β-TCP	1200 °C	~250	400	75 or 50	A	Schmidleithner et al. [55]	Grid and Kagome structure
**SL**	45S5 Bioglass^®^	950 °C	307	700–400	~60	A	Thavornyutikarn et al. [56]	Diamond-like structures
**SL**	CaSiO_3_–CaMgSi_2_O_6_	1100 °C	~500	~500	57–85	B	Elsayed et al. [57]	Diamond, kelvin and cubic structures
**SL**	Ca_3_−xM_2_x(PO_4_)_2_ (M = Na, K)	900–1400° C	500	50–750	70–80	B	Putlyaev et al. [58]	Kelvin structure
**SL**	β-TCP	1150 °C	1000	600–800	45	A	Weiguo et al. [59]	Spherical and cylindrical
**DIW**	Ca_3_SiO_5_	Setting reaction	200 (minimum)	200 (minimum)	60–65	C	Yang et al. [60]	Logpile*
**DIW**	Ca_7_Si_2_P_2_O_16_	1400 °C	1000	200 (minimum)	Up to 86	C	Uo et al. [61]	Logpile
**DIW**	Mesoporous bioactive glass/β-TCP	1100 °C	250	400	58	C	Zhang et al. [62]	Logpile
**DIW**	Ca_2_O_4_Si/CaSO_4_	Setting reaction	450	350	67	D	Pei et al. [63]	Logpile
**DIW**	Ca_2_MgSi_2_O_7_	1350 °C	450	400	65	D	Wang et al. [64]	Logpile
**DIW**	Cu, Fe, Mn, Co-doped bioactive glass	1300°C	500	250	<50	D	Liu et al. [65]	Logpile
**DIW**	Sr doped Ca_2_ZnSi_2_O_7_ /Al_2_O_3_	1250 °C	540	450–1200	50–70	D	Roohani et al. [66]	Logpile
**DIW**	45S5 bioactive glass	1050 °C	~250	287–820	60 to 80	C	Eqtesadi et al. [67]	Logpile
**DIW**	CaSiO_3_-CaMgSi_2_O_6_	1100 °C	320	390	68–76	D	Elsayed et al. [68]	Logpile
**SLS**	13-93 bioactive glass	700 °C	1000	1100	50	E	Kolan et al. [69]	Cubic
**SLS**	13-93 bioactive glass	695 °C	1000	1000	50	E	Kolan et al. [70]	Cubic
**SLS**	Ca_2_MgSi_2_O_7_	715–914 °C	2000	1000	<20	E	Shuai et al. [71]	Cubic
**SLS**	β-TCP/58S bioactive glass	No post treatment	1100	1500	56.04	F	Liu et al. [72]	Cubic
**SLS**	HA/β-TCP	No post treatment	800	1000	70.1	F	Gao et al. [73]	Cubic
**SLS**	45S5 bioglass	No post treatment	~3000	2000 × 2000 × 5000	~15	F	Liu et al. [74]	Channels
**SLS**	58S Bioactive glass/graphene	No post treatment	~1000	800	~50	F	Gao et al. [75]	Cubic

^+^ The finish quality is ranked based on the (i) porosity and roughness of the struts, (ii) differences between the computer model and print (not due to the sintering which results in a uniform volumetric shrinkage), and (iii) strut uniformity. Rank A: Scaffolds with solid microstructure (<1% porosity) and uniform struts (a size variation of ~20–50 µm), Rank B: Scaffolds with uniform struts that contain scattered micropores, Rank C: Scaffolds with solid microstructure and slight size variation in struts (~200 µm), Rank D: Scaffolds with microporous struts with slight size variation in struts, Rank E: Scaffolds with highly microporous and rough struts, or with nonuniform struts(size variation >300 µm), Rank F: Scaffolds with highly microporous, rough, and nonuniform struts. * For scaffolds fabricated by DIW technique, pore size is considered as the distance between deposited filaments (struts) on an x-y plane. Since in DIW technique, 3D constructs are generated by stacking of 2D layers that consist of filaments arranged in 2D directions, the definition of the pore is a space between the intersections of the filaments in three stacked layers. The pore size in z-direction always equals to the layer thickness (nozzle diameter), and in x-y direction equals to distance between adjacent filaments. Therefore, pores in scaffolds generated by DIW have a unique 3D geometry which is labeled as logpile.
